# Intrapartum caesarean delivery and childhood BMI trajectories in relation to the infant gut microbiome in the VDAART prospective birth cohort

**DOI:** 10.1016/j.ebiom.2026.106347

**Published:** 2026-06-26

**Authors:** Zheng Sun, Tong Wang, Jessica A. Lasky-Su, Augusto A. Litonjua, Scott T. Weiss, Jorge E. Chavarro, Yang-Yu Liu

**Affiliations:** a Channing Division of Network Medicine, Department of Medicine, Brigham and Women’s Hospital and Harvard Medical School, Boston, MA, USA; b Division of Pediatric Pulmonary Medicine, Golisano Children’s Hospital at Strong, University of Rochester Medical Center, Rochester, NY, USA; c Department of Epidemiology, Harvard T.H. Chan School of Public Health, Boston, MA, USA; d Department of Nutrition, Harvard T.H. Chan School of Public Health, Boston, USA

**Keywords:** Caesarean delivery, Gut microbiota, Childhood obesity, Childhood overweight, VDAART, GMPT

## Abstract

**Background:**

The rising global health crisis of childhood overweight and obesity is potentially influenced by caesarean delivery (CD), but it remains a subject of ongoing debate. The gut microbiome, which is affected by delivery mode and can impact body weight, might play a role in this issue. However, the complex relationship between them remains poorly understood.

**Methods:**

We analysed data from a randomised, double-blind, placebo-controlled trial VDAART cohort, including BMI percentiles from 683 children aged 2–8 years and 1672 stool samples collected between 3 months and 5 years (all data in this study were collected between May 2010 and February 2018). To evaluate how CD relates to BMI trajectories, we conducted permutation testing and discussed the effect of confounding factors. We then used PERMANOVA, random forest classification, and Generalised Microbe Phenotype Triangulation (GMPT) to explore the role of the gut microbiota in mediating this relationship.

**Findings:**

Compared with vaginal delivery, intrapartum CD (iCD) rather than antepartum CD (aCD) was associated with a higher BMI percentile trajectory (Δ = 31.8%; 95% CI, 16.25%–47.55%; P = 0.001, Permutation test), and this was observed only among female children. Moreover, delivery mode was significantly associated with early-life gut microbiota, with effects also limited to females (F = 2.15 and 2.47 at months 3–6 and at age 1; P = 0.035 and 0.007, PERMANOVA). Random Forest models using early microbiota data can predict later overweight/obesity, performing best among iCD-born females (AUROC = 0.88; 95% CI, 0.83–0.94 for age 2), indicating an optimal intervention window before age one. Finally, GMPT identified 24 early-life taxa potentially mediating iCD-related overweight/obesity risk (11 preventive; 13 permissive), including *Bacteroides ovatus*, *Bifidobacterium bifidum*, *Clostridium leptum*, *Eggerthella lenta, etc*.

**Interpretation:**

Our results indicate that CD types and children’s sex are key factors in this interaction, offering a possible explanation for the ongoing debate about whether CD is linked to childhood overweight/obesity, and providing valuable insights for future intervention strategies.

**Funding:**

This work was supported by the 10.13039/100000002National Institutes of Health.

**Research in context**

**Evidence before this study**

The rising global rates of childhood obesity and caesarean deliveries have prompted extensive research into a potential link between the two, but previous studies have yielded conflicting results. While it is known that the mode of delivery impacts an infant's early gut microbiome and that the gut microbiome influences body weight, the precise relationship between caesarean delivery, gut bacteria, and long-term weight development remains poorly understood. Existing human studies examining this relationship often lacked comprehensive, long-term gut microbiome data. Furthermore, studies that did include infant microbiome data rarely distinguished between the different circumstances leading to a caesarean section (such as whether labour had started before the surgery) or thoroughly investigated how the child's sex might influence the outcomes.

**Added value of this study**

By analysing a large, diverse group of mother-child pairs followed over several years, we discovered that not all caesarean deliveries carry the same risk. Specifically, only intrapartum caesarean deliveries (those performed after labour has already begun) were linked to a higher risk of childhood overweight and obesity. Notably, this increased risk was exclusively observed in female children. We also found that the mode of delivery significantly alters the infant's gut microbiome during the first year of life, and this disruption is primarily seen in females. Using predictive models, we demonstrated that these early microbial changes can strongly forecast whether a child will become overweight later in life. Finally, we identified 24 specific early-life gut bacteria that likely play a role in either promoting or preventing this excess weight gain.

**Implications of all the available evidence**

Our findings help explain why previous research on caesarean delivery and childhood obesity has been so contradictory: the specific circumstances of the surgery and the child's sex are critical missing pieces of the puzzle. Understanding that intrapartum caesarean delivery disrupts the early gut microbiome in ways that promote weight gain, particularly in girls, improves our understanding of the biological mechanisms behind early-onset obesity. This highlights the first year of life as a vital, optimal window for early intervention. The specific promotive and preventive bacteria identified in our study offer promising new targets for future clinical strategies, such as customised probiotics or precision diagnostics, aiming to restore a healthy gut microbiome and prevent obesity in at-risk children.

## Introduction

Childhood overweight and obesity are fast-growing public health concerns worldwide, with an estimated 39 million children under 5 years affected in 2020.^1^ Children who are overweight or obese are more likely to remain so into adulthood, which increases their risk of developing chronic diseases like cardiovascular disease, type 2 diabetes, certain respiratory diseases, and some cancers.^2^ As a life-saving practice for both mothers and infants, caesarean delivery (CD) has been increasingly adopted around the world. The debate regarding whether CD can increase the risk of childhood obesity is still ongoing. Some studies found no significant association between delivery mode and BMI z-scores, overweight, or obesity in children,^3^, ^4^, ^5^, ^6^ while others reported ambiguous results.^7^^,^^8^ Conversely, other studies reported that BMI z-scores and the risk of being overweight or having obesity were higher in children delivered by CD compared to vaginal delivery (VD).^9^, ^10^, ^11^, ^12^, ^13^, ^14^, ^15^, ^16^ These conflicting findings underscore the urgency of clarifying whether CD indeed influences childhood BMI development. If yes, identifying the potential contributing factors will pave the way for developing effective intervention strategies.^17^

Delivery mode has been consistently shown to impact the infant’s gut microbiome.^18^, ^19^, ^20^ In addition, alterations in the gut microbial composition have been linked to obesity.^21^, ^22^, ^23^ Therefore, piecing together the puzzle formed by these findings and understanding the relationships among CD, the gut microbiome, and childhood overweight/obesity holds potential to provide valuable insights for tackling this puzzle. However, despite evidence pointing to the significant influence of delivery mode on the gut microbiome of infants and the critical role of the gut microbiome in childhood overweight/obesity, the relationship between delivery mode and childhood overweight/obesity remains inconclusive.^24^ The existing human cohorts focusing on this question lacked large-scale gut microbiome data,^10^ whereas the infant cohorts that had the gut microbiome data, such as TEDDY^25^ and ECAM,^26^ did not thoroughly discuss the correlation between CD, gut microbiome, and childhood overweight/obesity, nor did they delve into detailed discussions of the circumstances leading to CD. This information is needed for the development of effective intervention strategies, especially those targeting the gut microbiome.

**Fig. 1:**
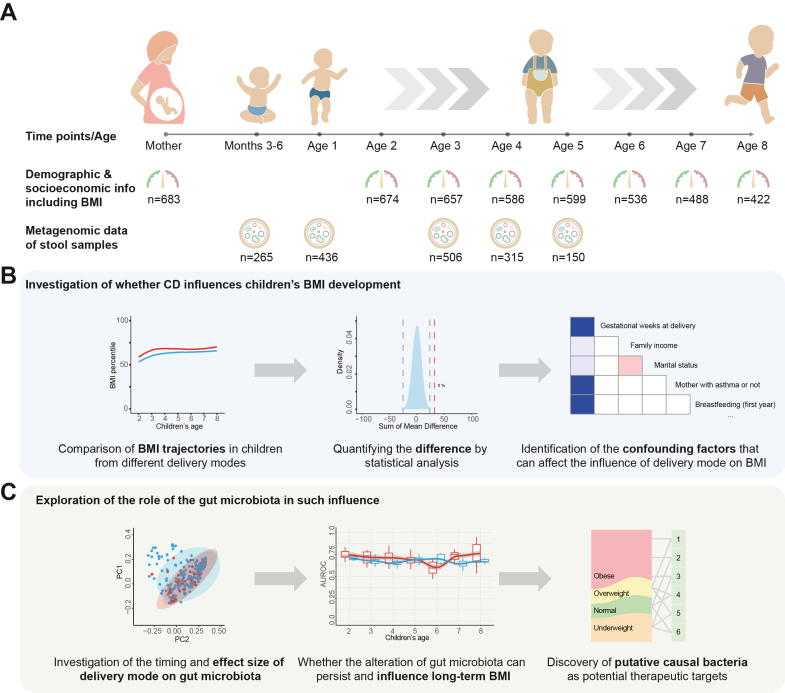
**Experimental design and analysis workflow.** (**A**) We accessed metagenomic sequencing data for 1672 samples from 683 mother-child pairs in the longitudinal VDAART cohort, along with their demographic and socioeconomic information. Our primary objective was to explore whether CD influences the development of children’s BMI. (**B**) We began by statistically comparing BMI percentile trajectories among children from different delivery modes. Subsequently, we identified factors that may affect how delivery mode influences BMI development. (**C**) Next, to assess the role of gut microbiota in the impact of delivery mode on BMI development, we examined how CD affects children’s gut microbiota. We then evaluated whether such changes in the gut microbiota could indeed affect long-term BMI development in children. Ultimately, we identified a causative bacterium associated with changes in BMI development following CD using GMPT.

Our study aims to decipher the role of the gut microbiome in connecting CD and childhood overweight/obesity. The ongoing debate regarding the relationship between CD and childhood overweight/obesity is primarily due to a lack of systematic analysis of factors that can modify the impact of CD on childhood overweight/obesity. For example, various factors, such as maternal obesity, lifestyle factors, children’s sex (biological sex at birth from the birth record), and genetics, have been reported to contribute to the intricate association in both human^27^ and animal studies.^28^ Therefore, we first examined the association of delivery mode with the BMI-percentile trajectory of children (from ages 2 to 8) in the Vitamin D Antenatal Asthma Reduction Trial (VDAART, Fig. 1a) and discussed the potential influence of confounding factors (Fig. 1b). Second, we investigated the correlations between the gut microbiome and Childhood Overweight/Obesity associated with Caesarean delivery (COOC). Based on the discovery of a significant association of delivery mode on children’s early-life gut microbiome, we further identified the specific time window during which delivery mode can relate to the gut microbiome, and whether such influence can persist throughout childhood up to age 8 to predict BMI development (Fig. 1c). Finally, we discovered a list of microbial taxa that may play a crucial role in COOC. This study provides a deeper understanding of the pathophysiology of childhood overweight and obesity, establishing a theoretical foundation for designing diagnostic and therapeutic clinical strategies.

## Methods

### Ethics

The study involved subjects who were the offspring of participants in VDAART, which is a multi-location, randomised, double-blind, placebo-controlled trial in the United States. VDAART aimed to investigate the preventive effects of Vitamin D supplementation during pregnancy on asthma and other allergies in the child (NCT00920621).^29^ Participants provided written consent prior to the study and the study protocol was strictly reviewed and approved by the Institutional Review Boards (IRB) at all participating clinical locations and the data coordinating centre. The participating clinical locations include Boston Medical Centre, Washington University in St. Louis, and the Kaiser Permanente Southern California Region in San Diego. The entire multi-site study and data coordination were conducted under a centralised IRB protocol approved by the Mass General Brigham Human Research Committee at Brigham and Women’s Hospital (Primary Protocol No. 2009P000557). The study was conducted in strict adherence to the ethical principles set forth in the 2024 Declaration of Helsinki.

### Sequencing and profiling of bacterial 16S rRNA

Stool samples in the VDAART study were sequenced using the 16S rRNA hypervariable region 4 (V4, 515F-806R) on the Illumina MiSeq platform (San Diego, CA) performed at Partners Healthcare Personalised Medicine (Boston, MA).^30^ To profile all 1672 samples’ raw reads, we used demultiplexing, quality filtering, and trimming tools provided by QIIME 2 (version 2022.8, RRID: SCR_021258).^31^ The clean reads (averaging 51,935 ± 24,962, ranging from 23,983 to 179,820) were subsequently denoised and clustered using *dada2 v1.22.0* (RRID: SCR_008205), and classification was performed with a pre-trained machine-learning-based classifier from the Silva database (release 138, RRID: SCR_006423). A total of 1384 Amplicon Sequence Variants (ASVs) were finally identified across all the samples. To evaluate potential environmental and reagent contamination, 21 negative controls (sequenced alongside different batches of the VDAART stool samples) were included. Taxa enriched in these negative controls were cross-checked against the key predictive and putative causal taxa identified in our downstream models, and the absence of overlap confirmed that our main biological signals were not caused by contamination artefacts. No rarefaction was conducted on the ASV table.

### Statistics

#### BMI trajectory modelling and permutation analysis

To model the progression of children’s BMI (percentile), we employed LOWESS (Locally Weighted Scatterplot Smoothing) fitting and the mean value, using the *ggplot2 v4.0.3* package (RRID: SCR_014601) in R v4.5.2 (The R Project for Statistical Computing, RRID: SCR_001905). For microbiome data analysis, the UniFrac distance matrices were calculated at the ASV level, utilising QIIME 2 (version 2022.8, RRID: SCR_021258).^31^

To determine if the disparities in BMI percentiles between the delivery modes were statistically significant, we assessed the probability of observing the differences between the two groups due to random chance. Specifically, we conducted a permutation analysis by randomly shuffling the BMI percentiles within each age group, repeating this process 10,000 times. This random shuffling allowed us to simulate a scenario where there was no inherent relationship between BMI percentiles and delivery mode. After each shuffle, we calculated the mean differences between the trajectories of the two groups, yielding a distribution of aggregated mean differences expected purely by chance. We then used a density plot for visualisation, which provides insights into the distribution's spread and shape, highlighting the range of mean differences that occur by chance. To determine the probability of observing a mean difference equal to or greater than the one observed in our study, we finally calculated the proportion of shuffled mean differences that surpassed our observed difference and then divided it by the total number of iterations (10,000). We define significance as 1/100 probability (0.01), meaning the difference being a certain number between vaginally delivery and CD is only 1% among all the 10,000 possibilities. The factors, including maternal gestational diabetes (n = 35), maternal age (>35, n = 52), intrauterine growth retardation (n = 20), preeclampsia (n = 34), and pregnancy-induced hypertension (n = 50), were excluded from this analysis due to their subgroup sizes constituting less than 10% of the overall population. The influence of factors such as recruitment site, education level, marital status, and family income, etc., on microbiome data was then evaluated using PERMANOVA (adonis2 in the *Vegan 2.7-3* package, RRID: SCR_011950).

### Linear mixed-effects model

To further test the longitudinal association between delivery mode and BMI percentiles and to validate our non-parametric trajectory visualisations, we employed a Linear Mixed-Effects Model (LMM). This approach accounts for the intra-subject correlation of repeated measurements across the 8-year follow-up period. Using the *lme4 v1.0* package (RRID: SCR_015654) in R, the longitudinally assessed BMI percentile was set as the continuous dependent variable, and a random intercept was included for each subject (child ID). To address potential confounding, the models were adjusted for key fixed-effect covariates, including the child's exact age, child sex, maternal race, and maternal asthma status. The primary predictor, delivery mode, was first evaluated as a broad category (overall CD vs. VD). Subsequently, we evaluated delivery mode by its subtypes (iCD, aCD, and VD), using VD and aCD as reference baselines in separate iterations.

### Prediction of overweight status using random forest

To determine whether the gut microbiota during infancy could serve as a predictor of long-term BMI development in children, we employed a Random Forest classifier (*randomForest v4.7-1.2* package in R with default parameters, RRID: SCR_015718). Specifically, we sought to use microbial data obtained from children at months 3–6 (or age 1) to predict their overweight status from age 2 to 8. Notably, there is substantial longitudinal overlap among these time points. For instance, 224 out of the 265 infants (84.5%) sampled at months 3–6 also provided stool samples at age 1. We emphasise that our goal is not to provide precise predictions of overweight status at various ages, but rather to examine the feasibility of using early life microbiome data as a determinant of overweight status from age 2 to age 8. To this end, we used the *randomForest v4.7-1.2* and *caret v7.0-1* packages (RRID: SCR_006260) in R with 10-fold cross-validation to compare predictive performance. To optimise predictive performance, systematic hyperparameter tuning was embedded within the cross-validation process. We used the train function with a tuneLength of 3, allowing the model to perform a grid search to optimise the mtry by maximising the Area Under the Receiver Operating Characteristic curve (AUROC). Furthermore, to ensure the stability of our predictions, this entire 10-fold cross-validation and tuning pipeline was repeated 10 independent times for each condition, and the maximum AUROC across the cross-validation folds was used to represent predictive power.

### Identification of putative causal taxa using GMPT

To elucidate the potential causal relationship between the gut microbiota and COOC, we applied GMPT^32^ to identify microbial taxa that act as preventive (inhibitory) or permissive (promotive) agents with respect to BMI, based on early-life gut microbiota profiles. This method has been previously validated in both murine models and human datasets, demonstrating its efficacy as a tool for pinpointing microbes linked to specific pathogens or phenotypic profiles. In our study, GMPT partitions microbiome samples into phenotype groups based on BMI percentiles (underweight, normal weight, overweight, and obesity). Differential abundance analysis is then performed for each phenotype pair to identify a pool of differentially abundant species using standard tools. A taxon was considered significant in a pairwise comparison if it achieved an unadjusted P < 0.05 (the Wilcoxon rank-sum test as the primary test, with ALDEx2 and Linear Model as Supplementary Methods). To determine the directionality of these effects, we evaluated abundance trends across BMI categories: taxa consistently depleted in higher-BMI groups relative to lower-BMI groups were designated as preventive (inhibitory), whereas those consistently enriched in higher-BMI groups were designated as permissive (promotive). To combine evidence and ensure robustness within our specific dataset, we utilised a frequency-based aggregation approach rather than relying on a single pooled statistical test. These differentially abundant species are subsequently ranked by their frequency of occurrence across all 42 pairwise phenotype comparisons (6 pairwise comparisons across 7 time points), in descending order. The underlying hypothesis of the method is that species that are differentially abundant in most pairwise phenotype comparisons and that demonstrate a strong negative (or positive) correlation with the abundance of a particular pathogen (or phenotype) are the putative causal species. In our study, we replaced the pathogen variable with children’s BMI and focused solely on children born via CD. By doing so, we ensured that the identified potential causal elements are relevant only to COOC. A taxon was identified as a candidate causal microbe only if it exhibited significant differential abundance in at least 10 out of the 42 comparisons. For exact statistics, we reported the aggregate frequency score and taxonomic classification in Supplementary Table S1. Based on the binomial distribution, assuming a single-test false positive rate of 0.05, the probability of a taxon reaching statistical significance in 10 or more comparisons purely by chance is approximately 2.5e–5. This stringent frequency requirement acts as a robust intrinsic filter, effectively controlling the global Type-I error rate and ensuring the reliability of the identified preventative and permissive taxa.

### Role of funders

The funders did not play any role in study design, data collection, data analysis, interpretation, or writing of the manuscript.

## Results

### Participants and data collection

The VDAART cohort is a randomised, double-blind, placebo-controlled trial that includes 881 mother-child pairs across the United States and assesses the impact of high-dose vitamin D supplementation during pregnancy on the development of asthma and allergies in offspring. Recruitment started in 2009, and follow-up is ongoing. After delivery, the VDAART study followed 876 children using quarterly over-the-phone health questionnaires and annual in-person visits. In these follow-ups, we further selected 683 children with relatively complete BMI data (BMI available at least two time points between ages 2 and 8; Fig. 1a). The demographic, socioeconomic, and metagenomic data from these children were subsequently used in the statistical analysis of this study.

We accessed the 16S rRNA sequencing data of a total of 1672 stool samples collected at various ages: months 3–6 (n = 265), age 1 (n = 436), age 3 (n = 506), age 4 (n = 315), and age 5 (n = 150). These samples comprised all the samples available from the 683 children in the VDAART study. Stool was not collected if the mother or child had used antibiotics in the past 7 days. The maternal participants (n = 683) comprise an ethnically and racially diverse group, with 27% identifying as Hispanic or Latino and 73% as non-Hispanic or Latino (Table 1). In terms of racial distribution, the cohort includes 39% white, 45% black, and 16% individuals of other races. The cohort also demonstrates a diverse range of family incomes, with 31% earning less than 30 k/year, 33% earning between 30 k and 100 k/year, and 12% earning more than 100 k/year. Pre-pregnancy BMI data show 55% of participants fall into the category of overweight, while education levels indicate that 57% have pursued at least some college education. In the context of health status, asthma is present in 41% of mothers. Delivery and early-life nutrition data show that most of their children (69%) were delivered vaginally, and during their first year of life, more than half were breastfed (52%), and the majority were given formula (85%). Furthermore, 52% of the mothers in this study were randomised to receive high-dose vitamin D supplementation during their pregnancies. In addition, the health status of the mother-child pairs that are relevant to CD during pregnancy (e.g., preeclampsia, intrauterine growth restriction, and children’s birth weight) was also recorded. All the above clinical covariates, which might be associated with gut microbiome or body weight, were considered potential confounders in the statistical analysis.

**Table 1:** Characteristics of the 683 mother-child pairs in this study.

Characteristic		Characteristic	
**Recruitment site**		**Children’s sex**	
Boston	194 (28%)	Female	321 (47%)
St Louis	270 (40%)	Male	362 (53%)
San Diego	219 (32%)	**Children’s ethnicity**	
**Family income**		Hispanic or Latino	223 (33%)
High level (>100 K/year)	82 (12%)	Not Hispanic or Latino	460 (67%)
Middle level (50K–100 K/year)	143 (33%)	**Children’s asthma status (at age 3)**	
Low level (<50 K/year)	294 (31%)	Yes	109 (16%)
Unknown	164 (24%)	No	569 (83%)
**Maternal education level**		**Children’s race**	
College graduate or Graduate school	233 (34%)	Black or African American	340 (50%)
Some college	160 (23%)	White	218 (32%)
High school or technical school	198 (30%)	Other	125 (18%)
Less than high school	92 (13%)	**Gestational weeks at delivery**	
**Marital status**		Preterm (<37 weeks)	139 (20%)
Not Married	356 (52%)	Term ( ≥ 37 weeks)	543 (80%)
Married	308 (45%)	**Children’s birth weight**	
Divorced	19 (3%)	Overweight (>4 kg)	50 (7%)
**Maternal ethnicity**		Normal weight (2.5–4 kg)	576 (84%)
Hispanic or Latino	182 (27%)	Underweight (<2.5 kg)	57 (9%)
Not Hispanic or Latino	501 (73%)	**Intrauterine growth retardation**	
**Maternal race**		Yes	20 (3%)
Black or African American	308 (45%)	No	663 (97%)
White	267 (39%)	**Preeclampsia**	
Other	108 (16%)	Yes	34 (5%)
**Maternal overweight status**		No	649 (95%)
Overweight	379 (55%)	**Formula in the first year**	
Normal	216 (32%)	Yes	582 (85%)
Unknown	88 (13%)	No	96 (14%)
**Maternal gestational diabetes**		Unknown	5 (1%)
Yes	35 (5%)	**Breastfeeding in the first year**	
Normal	648 (95%)	Yes	355 (52%)
**Maternal age**		No	323 (47%)
Advanced (>35)	52 (8%)	**Commencement of labour**	
Normal (<35)	631 (92%)	Induction	205 (30%)
**Mother with asthma or not**		Spontaneous	401 (59%)
Yes	277 (41%)	Scheduled CD	77 (11%)
No	406 (59%)	**Delivery mode**	
**Vitamin D Treatment for mothers**		Vaginal delivery (VD)	472 (69%)
High dose (4400 IU)	352 (52%)	Caesarean delivery (CD)∗	211 (31%)
Low dose (400 IU)	331 (48%)		
**Pregnancy-induced hypertension**		∗ Intrapartum CD (iCD)	134
Yes	50 (7%)	∗ Antepartum CD (aCD)	77
No	633 (93%)		

Data are given as numbers (and percentages) of individuals. In addition to children’s characteristics, their mothers' information, such as sex, ethnicity, race, and disease status, is also included. All cases that entered labour, whether spontaneously or induced, and ultimately required a CD due to conditions such as preeclampsia, intrauterine growth restriction, etc., were classified as iCD.

### A significant association between intrapartum CD and children’s BMI

**Fig. 2:**
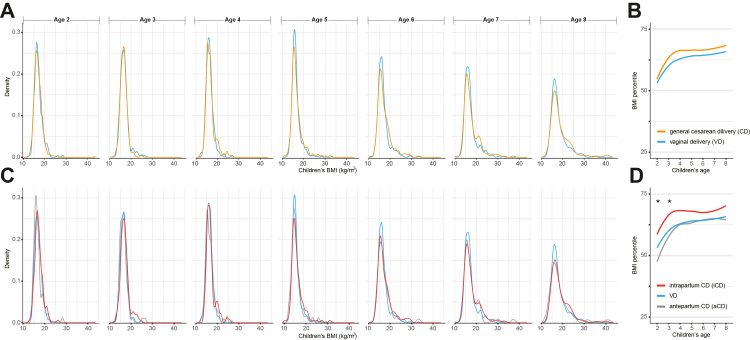
**Comparison of BMI and BMI percentile trajectories by delivery mode in children from age two to eight.** (**A**) The distribution of children’s BMI between children born via general caesarean delivery (CD, n = 211) and vaginally delivery (VD, n = 472) from ages 2 to 8. (**B**) The trajectory of BMI percentiles in children from the VDAART study (n = 683), aged 2 to 8, categorised by mode of delivery. In this comparison, all cases that had a caesarean section, regardless of whether it was antepartum or intrapartum, were categorised as CD and compared with VD. The classification adheres to CDC guidelines. The analysis uses the LOWESS method to generate curve-fitting lines. (**C**) The distribution of children’s BMI percentiles between children born via iCD (n = 134), aCD (n = 77), and VD (n = 472) at different ages. (**D**) The trajectory of BMI percentiles between intrapartum caesarean delivery (iCD), antepartum caesarean delivery (aCD), and VD. In this comparison, all cases that entered labour, whether spontaneously or induced, and ultimately required a CD due to acute intrapartum indications such as failure to progress or foetal distress, were classified as iCD.

To elucidate potential associations between CD and the risk of childhood overweight/obesity, we first investigated the trajectory of children’s BMI in the VDAART study (n = 683). Specifically, we initially compared the BMI distribution (Fig. 2a) from age 2 to age 8, between children born via CD (n = 211) and VD (n = 472). However, the difference is not readily observable between the two groups. To better visualise and understand BMI development, we then examined BMI percentile trajectories (Fig. 2b) and quantified the differences. At each age, we computed the mean BMI percentile for each delivery mode and calculated the difference between them. Then we summed across all time points from ages 2 to 8 to obtain the cumulative mean BMI percentile difference between the two delivery modes, denoted as Δ(CD,VD). We found that Δ(CD,VD)=16.94% (95% CI, 3.46%–30.88%). To quantify the statistical significance of this result, we computed the empirical P-value, denoted as P(CD,VD), representing the probability of observing Δ(CD,VD)=16.94% purely by chance (Methods). We found that P(CD,VD)=0.008 (Permutation test).

To further understand whether CD types can impact children’s BMI development, we next divided the CD group into two subgroups based on the circumstance leading to CD: antepartum CD (aCD, n = 77), which defined as cases with C-section performed before labour begins, and intrapartum CD (iCD, n = 134) defined as cases that entered labour, whether spontaneously or induced, and ultimately required a C-section due to acute intrapartum indications such as failure to progress or foetal distress. When comparing the BMI percentile trajectories of iCD and VD, we found that Δ(iCD,VD)=31.8% (95% CI, 16.25%–47.55%) and P(iCD,VD)<0.001 (Permutation test). By contrast, when comparing the BMI percentile trajectories of aCD and VD, we found that Δ(aCD,VD)=−11.08% (95% CI, −31.38% to 9.02%) and P(aCD,VD)=0.141 (Permutation test). This suggests that the significance of CD and VD in BMI trajectories is mainly contributed by iCD instead of aCD. In addition to the empirical P-value calculation, we performed Student’s t-test to compare the BMI percentile differences across delivery modes at each time point. We found that only when comparing iCD vs. VD yielded statistically significant results for ages 2 and 3 (P= 0.042 and 0.043, respectively, Student's t-test, Fig. 2d). This indicates that without differentiation of CD subtypes, the mode of delivery does not significantly influence BMI development.

To validate these observational trajectory differences and account for repeated measures, we further employed a linear mixed-effects model (LMM) adjusting for child sex, age, maternal race, and maternal asthma. Consistent with our permutation tests, when overall CD was compared to VD without subtyping, no significant association with longitudinal BMI percentiles was observed (Estimate = −2.31, P = 0.346, Linear Mixed-Effects Model). This confirms that CD, as a broad category, does not significantly affect BMI development. However, differentiating CD subtypes in the model effectively revealed the masked association. When setting VD as the baseline reference, being born via iCD emerged as a highly significant independent predictor of elevated BMI percentiles (Estimate = 6.81, P = 0.0183, Linear Mixed-Effects Model), whereas aCD showed no significant difference from VD (P = 0.1026, Linear Mixed-Effects Model). Furthermore, when aCD was used as the reference group, iCD remained a highly significant predictor of higher BMI percentiles (Estimate = 12.89, P = 0.0028, Linear Mixed-Effects Model).

### The effect of iCD on children’s BMI is sex-dependent

**Fig. 3:**
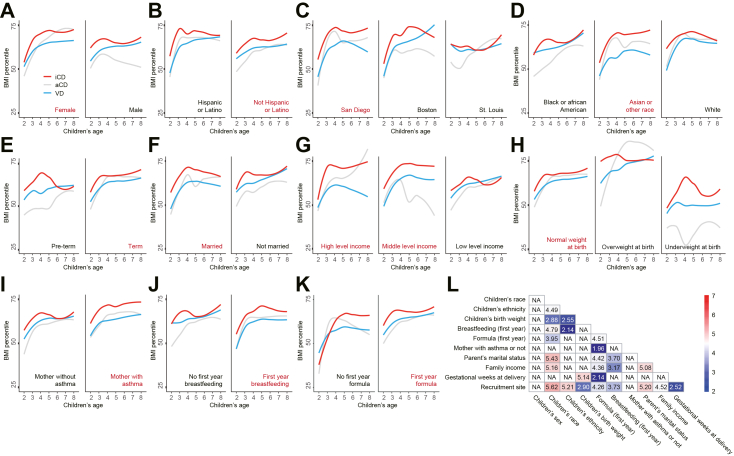
**Key confounding factors influencing the impact of iCD on children’s BMI development.** This figure illustrates the trajectories of children’s BMI percentiles from ages 2 to 8, differentiated by delivery mode (iCD in red, aCD in grey, VD in blue). Significant factors include children's sex (**A**, Female n = 321, Male n = 362), children’s ethnicity (**B**, Hispanic or Latino n = 223, Not Hispanic or Latino n = 460), recruitment site (**C**, Boston n = 194, St Louis n = 270, San Diego n = 219), maternal race (**D**, Black or African American n = 308, White n = 267, Other n = 108), gestational weeks at delivery (**E**, Term n = 543, Preterm n = 139), parent’s marital status (**F**, Not Married n = 356, Married n = 308, Divorced n = 19), family income (**G**, High n = 82, Middle n = 143, Low n = 294, Unknown n = 164), children’s birth weight (**H**, Normal n = 675, Overweight n = 50, Underweight n = 57), maternal asthma status (**I**, Yes n = 277, No n = 406), first-year breastfeeding (**J**, Yes n = 355, No n = 323, Unknown n = 5), and first-year formula feeding (**K**, Yes n = 582, No n = 96, Unknown n = 5). Subgroup labels highlighted in red indicate subgroups in which the difference in childhood overweight/obesity risk between delivery modes reached statistical significance (P < 0.001, Permutation test). Labels in black indicate subgroups in which the difference did not reach this threshold. (**L**) Heatmap of the Chi-square test statistics (χ^2^) assessing the pairwise associations among the significant categorical confounding factors for COOC. The colour intensity represents the magnitude of the χ^2^ statistic. NS indicates non-significant associations (P > 0.01, Chi-square test).

We then sought to explore whether the risk of iCD-associated childhood overweight/obesity is modified by key maternal and paediatric clinical and demographic covariates. Specifically, we incorporated demographic and socioeconomic information from the VDAART study (as detailed in Table 1) into the above analysis to identify factors that could significantly heighten/weaken the difference between the two BMI percentile trajectories. We found nine factors (among 23 factors in Table 1) that significantly influenced the COOC risk, including the children’s sex, race, birth weight, and ethnicity, the recruitment site, the family’s income, the first-year breastfeeding and formula feeding status, the maternal asthma status, the gestational weeks at delivery, and the parental marital status. For example, significant COOC risk were observed under the following conditions (P<0.001, Permutation test): female children (Fig. 3a), not Hispanic or Latino children (Fig. 3b), children born in San Diego (Fig. 3c), Asian and other race mothers (Fig. 3d), children born from term pregnancy (Fig. 3e), children from married couples (Fig. 3f), children from higher or middle-income families (Fig. 3g), children with normal (2.5 kg–4 kg) birth weight (Fig. 3h), children born to asthmatic mother (Fig. 3i), children with first-year breastfeeding (Fig. 3j) and formula feeding (Fig. 3k). Notably, maternal overweight status and children’s high birth weight do not further increase the COOC risk. This finding clarifies the impact of these two significant confounding factors on childhood overweight/obesity and advances the study of the pathological aetiology of COOC.

To further investigate the potential associations of COOC with various confounding factors, it was necessary to determine whether the factors above are independent. Accordingly, we conducted a Chi-squared test on all 10 of the above significant factors. The results suggest that these factors are not necessarily independent, as evidenced by the chi-squared test (Fig. 3l). Nonetheless, we identified the children’s sex as the only independent factor. These findings suggest that iCD is significantly associated with an increased risk of childhood overweight/obesity under specific circumstances, particularly among female children (Fig. 3a). As for other factors, it is challenging to assess their independent association with COOC without utilising another substantial large-scale cohort.

### Children’s gut microbiota is associated with COOC

Given the well-established evidence of the close relationship between the gut microbiota and body weight, we next explored the role of children’s gut microbiota in COOC to understand the mechanisms underlying the association between iCD and childhood overweight/obesity. We accessed high-throughput metagenomic sequencing data of 1672 children’s stool samples from the VDAART study, including 265 samples collected during months 3–6, 436 at age 1, 506 at age 3, 315 at age 4, and 150 at age 5 (Methods). After excluding influence from technical variations (such as sequencing batch and recruitment site), we identified age as the most significant factor influencing children’s gut microbiota composition (Permutational Multivariate Analysis of Variance (PERMANOVA) pseudo-F ratio = 130.93, P=0.001, PERMANOVA), followed by children’s overweight/obesity risk (F = 6.98, P=0.001, PERMANOVA), gestational age at delivery (F = 6.08, P=0.001, PERMANOVA), children’s high overweight/obesity risk (F = 5.67, P=0.001, PERMANOVA), and delivery mode (iCD vs. aCD vs. VD, F = 5.49, P=0.001, PERMANOVA), which emerged as the Top 5 factors among all the demographic and socioeconomic factors (Table 2). This suggests that not only BMI but also delivery mode are strongly associated with the gut microbiota, thereby highlighting the close relationship between COOC and the gut microbiota. Notably, the classification of children’s overweight/obesity risk here is determined by evaluating the overall trajectory of children’s BMI percentile from age 2 to age 8, following the recommendations established by the US Centres for Disease Control and Prevention (CDC). If a child is deemed overweight or obese at any follow-up visits between the ages of 2 and 8, they will be considered to have any occurrence of overweight/obese in our analysis. This applies to 387 out of the 683 children (56.6%) studied. Similarly, if a child is observed to be overweight or obese at least two follow-up visits between the ages of 2 and 8, they are classified as having **recurrent** overweight/obese in our analysis. This applies to 271 out of the 683 children (43.1%) studied.

**Table 2:** Effect size of clinical and demographic factors on children’s gut microbiota.

Factor name	Pseudo-F value	P-value
Children’s age	**130.93**	**0.001**
Any occurrence of overweight/obesity	**6.98**	**0.001**
Gestational weeks at delivery	**6.08**	**0.001**
Recurrent overweight/obesity	**5.67**	**0.001**
Delivery mode (iCD vs. aCD vs. VD)	**5.49**	**0.001**
Marital status	**5.27**	**0.001**
Commencement of labour	**4.30**	**0.001**
Maternal race	**4.18**	**0.001**
Maternal education level	**4.11**	**0.001**
Breastfeeding in the first year	**3.87**	**0.001**
Children’s ethnicity	**3.81**	**0.009**
Children’s asthma status	**3.61**	**0.002**
Children’s race	**3.59**	**0.001**
Delivery mode (CD vs. VD)	**3.47**	**0.001**
Family income	**2.43**	**0.002**
Maternal ethnicity	2.42	0.041
Vitamin D Treatment for mothers	2.36	0.045
Maternal age	2.27	0.041
Formula in the first year	2.21	0.027
Maternal overweight status	1.52	0.108
Maternal gestational diabetes	1.34	0.175
Children’s sex	1.13	0.263
Children’s birth weight	1.09	0.306
Mother with asthma or not	0.85	0.484

We investigated the effect size of all the factors in Table 1 on the infant gut microbiota (n = 1672 stool samples from 683 children) and sorted them by PERMANOVA pseudo-F ratio. Intrauterine growth retardation, Preeclampsia, Maternal gestational diabetes, and Pregnancy-induced hypertension were excluded from the analysis due to a very unbalanced sample size (with one group in the binary factor having less than 10% of the cohort). Significant factors (P < 0.01, PERMANOVA) were highlighted in bold.

### The association between children’s gut microbiota and delivery mode is also sex-dependent

**Fig. 4:**
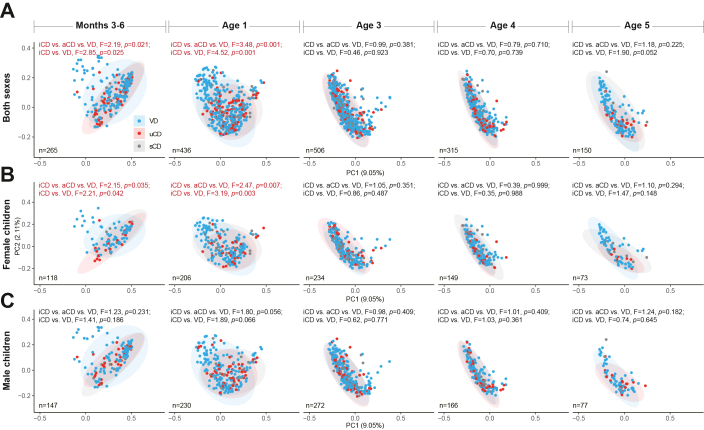
**The association between COOC and the gut microbiota.** PCoA plot of infant gut microbiota from months 3–6 to year 5 (total samples: months 3–6, n = 265; age 1, n = 436; age 3, n = 506; age 4, n = 315; age 5, n = 150), coloured by delivery modes and stratified by sex: both sexes (**A**), female children (**B**), and male children (**C**). Effect sizes of delivery mode are indicated by F values from PERMANOVA tests and are labelled in the figures. Significantly different results (PERMANOVA P-values < 0.05) are highlighted in red.

Considering the strong influence of age, we then conducted Principal Coordinates Analysis (PCoA) and PERMANOVA using the weighted UniFrac distance across all stool samples from different age groups. This allowed us to visualise and explore the relationship between gut microbiota and delivery mode while minimising the influence of age. The analysis revealed that delivery mode remains significant associated with the early-stage gut microbiota of children, particularly within the first year of life (F = 2.19, P=0.021, and F = 3.48, P=0.001, PERMANOVA, for months 3–6 and age 1 respectively, Fig. 4a). We next hypothesised that the sex-dependent occurrence of COOC, as observed in our previous statistical analysis (Fig. 3a), could be mediated by children’s gut microbiota. To test this hypothesis and further investigate the role of the gut microbiota in COOC, we conducted PCoA and PERMANOVA again under different children’s sexes and ages. Our results demonstrated that delivery mode significantly influenced the gut microbiota composition of female children, particularly in their early infancy (F = 2.15, P=0.035; F = 2.47, P=0.007, PERMANOVA, for months 3–6 and age 1, respectively, Fig. 4b), whereas no such association was observed in male children (F = 1.23, P=0.231; F = 1.80, P=0.056, PERMANOVA, for months 3–6 and age 1, respectively, Fig. 4c). These findings suggest that the gut microbiota is strongly associated with the onset of COOC, offering a mechanistic explanation for the sex dependency observed in COOC.

### Identifying the temporal window when delivery mode is associated with the gut microbiota

To further understand the relationship between COOC and gut microbiota, we asked two questions: (1) How long does the association of iCD persist on the gut microbiota during early childhood? (2) Are such changes in gut microbiota critical for long-term BMI development? To tackle the first question, we investigated the temporal window during which the delivery mode may influence the gut microbiota and found that the impact of delivery mode on gut microbiota composition persisted significantly at the end of the first year (Fig. 4a), particularly in female children.

**Fig. 5:**
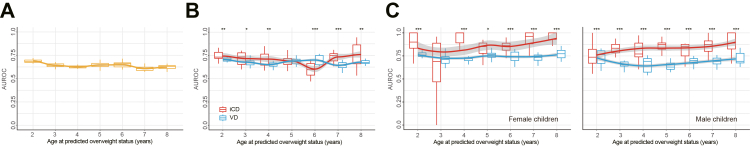
**Prediction of children’s overweight status using early-life gut microbiota.** (**A**) The performance of Random Forest models using metagenomic data of months 3–6 (n = 265) and age 1 (n = 436) to predict the children’s overweight status from age 2 to 8. (**B**) The AUROC of Random Forest models using metagenomic data from iCD born (n = 134) and vaginally born (n = 472) children (including data from both months 3–6 and age 1) in predicting the overweight status of children from age 2 to 8. We then stratified the early-life gut microbiota data by sex to illustrate their predictive performance for children’s overweight status in (**C**). Model performance, evaluated using metagenomic data from months 3–6 (n = 265) and at age 1 (n = 436), was incorporated for virtualisation. For all box plots, the central horizontal line represents the median, the lower and upper hinges of the box indicate the 25th and 75th percentiles (interquartile range, IQR), respectively, and the whiskers extend to the most extreme data points that are no more than 1.5 × IQR from the box. Each box plot is based on n = 10 independent iterations of 10-fold cross-validation. ∗, P < 0.05; ∗∗, P < 0.01; ∗∗∗, P < 0.001 (Student’s t-test).

To assess whether the gut microbiota during this temporal window plays a critical role in predicting long-term BMI development in children, we hypothesised that if such an influence exists, it would be reflected in the power of microbiome samples collected from this period in predicting children’s overweight status from ages 2 to 8. The higher the prediction power, the greater the potential impact. Based on this hypothesis, we used the Random Forest (RF) classifier with metagenomic data (the abundance of all identified taxa) of both months 3–6 and age 1 as features to predict children’s overweight/obesity status (dichotomised as overweight and obesity vs. others based on CDC guidelines) from age 2 to 8. The prediction power of the RF classifier was subsequently evaluated using the Area Under the Receiver Operating Characteristic curve (AUROC) from 10-fold cross-validation. Our analysis revealed that the predictive power of early-life microbiota (both months 3–6 and age 1) for predicting long-term overweight/obesity status tends to decrease over time, yet remains relatively high at age 8. For instance, the gut microbiota at 3–6 months was able to predict overweight/obesity status at age 2 with an AUROC of 0.68 (95% CI, 0.67–0.70) and decreased to 0.60 (95% CI, 0.59–0.62) by age 8 (Fig. 5a). In addition, the predictive performance varies between children born via iCD and those born vaginally, e.g., children born via iCD exhibit better overall performance of predictions (Fig. 5b), suggesting a stronger association between the gut microbiota and BMI development born via iCD. The above results suggest a potential mechanism for COOC: early-life alterations in the gut microbiota induced by iCD may increase the risk of overweight/obesity later in life.

We next incorporated sex as a variable in the predictive analysis, we observed that metagenomic data of: (1) vaginally born children of either sex exhibit relatively stable prediction performance for childhood overweight/obesity; (2) iCD born children of either sex exhibit better prediction performance; (3) Female children born via iCD exhibit the highest predictive power with AUROC reaching up to an average of 0.88 (95% CI, 0.83–0.94) (Fig. 5c). In summary, the first year of life represents a critical period where the mode of delivery is significantly associated with the gut microbiota, potentially impacting BMI development and exhibiting variations based on the children's sex.

### Discovery of the preventive and permissive microbial taxa in COOC

Given that early-life alterations in the gut microbiome induced by iCD may increase the occurrence of overweight or obesity later in life, we sought to explore whether an inferred causal relationship exists between specific taxa in children’s gut microbiome in early life and their childhood overweight/obesity status. To perform this computational causal analysis, we employed GMPT, which is based on the hypothesis that differential microbial taxa consistently appearing in pairwise “phenotype” comparisons and exhibiting strong negative (or positive) correlations with disease severity are putative causal microbial taxa that are preventive (or permissive). For children born via iCD, at each age from 2 to 8, we stratified them into four phenotype groups based on their BMI percentile (underweight, normal weight, overweight, and obesity as defined by the CDC) and then performed GMPT using their metagenomic data collected in early life (months 3–6, or age 1). We repeated the above analysis for children born via VD too (Methods).

**Fig. 6:**
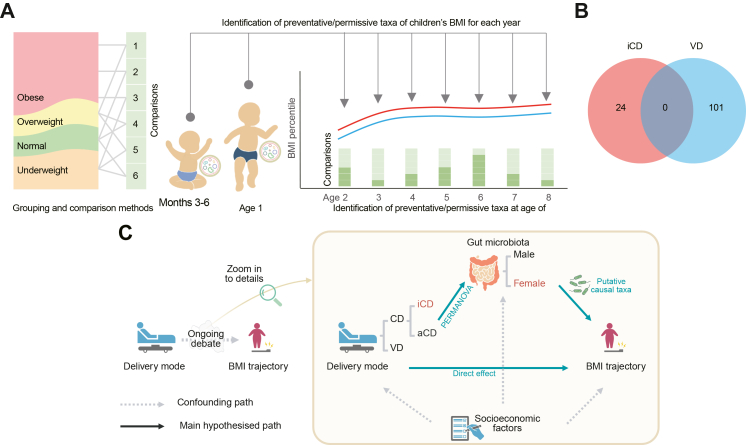
**Putative causal bacteria discovery of COOC.** (**A**) A schematic illustration of how the GMPT was applied: For each age between 2 and 8 years, metagenomic data from months 3–6 (n = 265) and age 1 (n = 436) were stratified into four groups: obese, overweight, normal, and underweight, by BMI percentiles, which were then pairwise compared to yield six comparisons. Ultimately, we inferred a list of potentially candidate causal taxa based on the number of significant results across 42 comparisons (6 comparisons across 7 time points). (**B**) Venn diagram illustrating the intersection of taxa inferred to cause childhood overweight/obesity between children born via iCD (n = 134) and those born vaginally (n = 472). (**C**) Conceptual model illustrating the hypothesised potential causal pathways linking delivery mode, gut microbiota, and childhood BMI trajectory.

In this analysis, we inferred the candidate causal taxa for childhood overweight/obesity based on the frequency of significant differences for each taxon across 42 comparisons (6 comparisons across 7 time points), setting a threshold of 10/42 (Fig. 6a). From this, we identified 24 and 101 microbial taxa, ranging from the phylum level to the Amplicon Sequence Variant (ASV) levels, in the early life microbiota (from stool samples at months 3–6 and age 1) that could potentially serve as critical taxa for COOC in children via iCD or VD, respectively (Fig. 6b, Supplementary Table S1). We next hypothesise that true putative causal bacteria of COOC should only appear in the iCD group because we assume that the microbial signature of children born via iCD with/without overweight will differ from that of VD children with/without overweight. This leads us to target the 24 taxa uniquely identified in the iCD group (Table 3). We found that among the 24 putative causal taxa we inferred, there were 17 ASVs (corresponding to nine species-level phylogenetic annotations and one family level annotation), 3 genera (*Gordonibacter*, *Megamonas*, and *Solobacterium*), and one each of phylum (Patescibacteria), class (Saccharimonadia), order (Saccharimonadales), and family (Selenomonadaceae). Among these, 11 were preventive and 13 were permissive for childhood overweight/obesity. Interestingly, most of these taxa (22 out of 24) have been associated with overweight or obesity in previous studies. For instance, taxa such as *Bacteroides ovatus*, *Bifidobacterium bifidum*, *Clostridium leptum*, *Eggerthella lenta*, *Evtepia gabavorous*, *Faecalibacterium prausnitzi*, *Lachnospiraceae bacterium*, *Parabacteroides distasonis*, *Gordonibacter*, *Megamonas*, *Solobacterium, Patescibacteria*, *Saccharimonadales*, *Saccharimonadia*, and *Selenomonadaceae* have been noted, yet they haven’t been well studied specifically in the context of childhood overweight/obesity or CD.

**Table 3:** Permissive and preventive taxa for COOC identified by using GMPT.

Taxonomic annotation	Phylogenetic level	Rank	Inhibitor (−) or promoter (+) to BMI	Reference
Lachnospiraceae bacterium	ASV	14	−	^27^
*Bifidobacterium bifidum*	ASV	13	+	^33^
*Bifidobacterium bifidum*	ASV	13	+	^33^
*Eggerthella lenta*	ASV	12	+	^34^
Lachnospiraceae bacterium	ASV	12	−	^27^
*Eggerthella lenta*	ASV	12	+	^34^
Lachnospiraceae bacterium	ASV	11	−	^27^
*Enterocloster lavalensis*	ASV	11	−	–
Lachnospiraceae bacterium	ASV	11	+	^27^
*Blautia hominis*	ASV	11	−	–
*Gordonibacter*	Genus	11	+	^35^
*Parabacteroides distasonis*	ASV	10	+	^36^
*Faecalibacterium prausnitzii*	ASV	10	−	^37^
*Bacteroides ovatus*	ASV	10	−	^38^
*Eggerthella lenta*	ASV	10	+	^34^
*Evtepia gabavorous*	ASV	10	+	^39^
*Bacteroides ovatus*	ASV	10	−	^38^
*Clostridium leptum*	ASV	10	+	^40^
*Megamonas*	Genus	10	−	^41^
Patescibacteria	Phylum	10	+	^42^
Saccharimonadales	Order	10	+	^43^
Saccharimonadia	Class	10	+	^44^
Selenomonadaceae	Family	10	−	^45^
*Solobacterium*	Genus	10	−	^46^

The taxa are ranked based on their likelihood of being causal (computationally inferred), defined as the number of times they reached statistical significance (P < 0.05, Wilcoxon test) across 42 differential analyses (7 time points × 6 group comparisons). Additionally, the table includes the taxonomic level of each taxon, indicates whether it acts as an inhibitor (−) or promoter (+) of BMI development, and notes whether previous studies have associated it with overweight, obesity, or BMI, along with the corresponding references. Multiple entries for the same taxonomic annotation represent distinct ASVs that map to the same species but possess different sequence compositions.

## Discussion

Despite evidence pointing to the significant influence of delivery mode on the gut microbiome of infants^19^ and the critical role of the gut microbiome in childhood overweight/obesity,^22^ the relationship between delivery mode and childhood overweight/obesity remains inconclusive.^24^ This greatly hinders the development of effective intervention strategies, especially those targeting the gut microbiome, in response to the escalating global public health concern of childhood overweight/obesity. Our findings illustrate that without differentiation of CD types, the mode of delivery is not significantly associated with BMI development, e.g., only children born via iCD have a significantly higher risk of being overweight or obese in their childhood. Moreover, the association between iCD and children’s BMI is sex-dependent, e.g., only female children exhibit a significantly increased risk of childhood overweight/obesity (Fig. 6c). Notably, sex is a factor that has been seldom discussed previously, aligning only with findings from a mouse model study.^28^ Additionally, CD types are not well discussed in newborn cohorts with microbiome data involved. Therefore, we believe that previous inconsistent observations may stem from the omission of CD types and sex as variables, while our analysis offers a new perspective to address the ongoing debate regarding the relationship between delivery mode and childhood overweight/obesity.

The distinct association of iCD, compared to aCD, with childhood BMI trajectories may be due to several potential factors. Unlike the controlled setting of an aCD, where membranes remain intact, and prophylactic antibiotic use is brief, iCD often involves prolonged rupture of membranes and extended intrapartum antibiotic treatment (e.g., for group B streptococcus prophylaxis or chorioamnionitis).^47^ This environment can facilitate abnormal ascending microbial colonisation despite heavy antibiotic pressure. Additionally, the combination of intense labour-induced inflammatory stress followed by surgical delivery creates a unique environment that may significantly disrupt the pioneer microbiome and early metabolic programming, leading to the increased occurrence of overweight/obesity observed solely in the iCD group.^48^ Clinically, the intense physiological stress and maternal exhaustion associated with an iCD can alter early feeding practices, such as reducing exclusive breastfeeding or shifting the initiation of solid foods. This nutritional shift frequently cascades from immediate clinical challenges unique to unplanned intrapartum surgical interventions, such as delayed mother-infant skin-to-skin contact, heightened postoperative pain, and the delayed onset of lactogenesis II.^49^ As a result, these infants exhibit a higher susceptibility to delayed breastfeeding initiation and a premature reliance on formula. Given that human milk uniquely provides essential substrates that promote the proliferation of beneficial pioneer taxa (such as Bifidobacterium species identified as key preventive microbes against overweight/obesity in our analysis), this iCD-driven disruption of early nutrition likely compounds the initial microbial perturbations, further exacerbating long-term childhood adiposity risk.^50^

On the other hand, the close association between CD, BMI, and gut microbiota has been further validated in this study. We discovered that the impact of CD on the gut microbiota has three notable characteristics. First, its association is confined to a short time window; specifically, we found that iCD only significantly relates to the gut microbiota before age 1. Regrettably, we lack microbiota data between the first and second year, preventing us from specifying the exact end of this time window. Secondly, this short-term influence exhibits sex specificity, echoing the sex dependency observed in COOC, which is unlikely to be coincidental given that the available stool samples maintained a balanced sex composition (as longitudinal sample missingness was not differential by sex), and besides age, delivery mode, and the occurrence of overweight are indeed among the top factors influencing the gut microbiota. Lastly, this short-term effect has long-term implications, influencing BMI development as evidenced by the high accuracy of using early-life (before age 1) gut microbiota to predict outcomes of childhood overweight/obesity (ages 2 to 8). These three points collectively underscore the strong association between COOC and infant gut microbiota, further supporting our hypothesis that children's gut microbiota plays a pivotal mediating role in COOC, paving the way for a deeper understanding of the risk factors (gut bacteria) that have therapeutic or intervention potential.

To design effective interventions to prevent COOC, it is crucial to establish the causal chain linking iCD, the gut microbiota, and childhood overweight/obesity, which is not possible without causal analysis. Although animal experiments support an interpretation of causal relationship rather than mere association, this has rarely been studied in humans.^28^ In our research, we first identified a critical time window in early childhood during which the mode of delivery relates to the gut microbiota and, consequently, long-term BMI development. Then, we employed GMPT to perform a computational causality analysis, and our findings revealed that 24 taxa play a pivotal role in COOC. Among the 24 taxa, *Lachnospiraceae bacterium* (4 ASVs), *B. bifidum* (2 ASVs), and *E. lenta* (2 ASVs) were phylogenetic annotations that appeared multiple times in the top 10 putative causal ASVs. Previous studies have already shown many significant associations between them and body weight or BMI. For instance, a mother-child cohort study revealed that birth mode and infant gut microbiota (particularly Lachnospiraceae) sequentially mediate the association between maternal prepregnancy overweight and childhood overweight.^27^ The underlying mechanism may involve Lachnospiraceae's capacity to produce long-chain fatty acids such as elaidate, which consequently facilitates diet-induced obesity.^51^

*B. bifidum*, either as a standalone intervention or in combination with other probiotics and/or dietary fibre, shows potential benefits for weight management and obesity-related outcomes.^33^^,^^52^ Notably, comparative studies have revealed a significant depletion of *B. bifidum* in the gut microbiota of obese individuals relative to their lean counterparts.^53^ Clinical evidence suggests that supplementation with *B. bifidum* can effectively reduce body weight and BMI in overweight and obese adult populations.^54^ As for *E. lenta*, it has also been found to be associated with obesity and type 2 diabetes.^34^ For example, its abundance is elevated in obese individuals compared to their lean counterparts within a Japanese cohort.^55^ Additionally, evidence indicates that *E. lenta* is consistently enriched in populations with obesity, demonstrating a positive correlation with increased adiposity and metabolic disorders.^56^ Despite the emerging evidence of *E. lenta* and *B. bifidum*‘s role in various metabolic and inflammatory conditions, the specific contributions of these bacteria to the metabolic health of overweight and obese individuals remain to be fully elucidated.^18^ Therefore, further investigations are necessary to clarify its role and evaluate its potential as a biomarker or therapeutic target.

Emerging evidence indicates that sex hormones, immunity, and the gut microbiome interact in a sex-specific manner to associate with metabolic health. Oestrogens, acting through receptors such as ERβ, generally promote Th2 and B-cell–mediated responses, enhance gut barrier integrity, and suppress low-grade inflammation, whereas testosterone exhibits more immunosuppressive effects.^57^, ^58^, ^59^ These hormones both shape—and are shaped by—the gut microbiota via the “microgenderome,” creating bidirectional feedback loops linked to obesity and metabolic syndrome.^60^ Within this framework, *B. bifidum* and *E. lenta* may exert sex-specific effects in obesity. For example, *B. bifidum*, known to strengthen epithelial barrier function, modulate NF-κB/IL-10 signalling, and generate beneficial metabolites such as SCFAs, may synergise with oestrogen-driven Th2 polarisation to reduce inflammation and adiposity, particularly in women.^61^ While these endocrine-microbiome interactions are predominantly characterised in adults, infants experience a transient, sex-specific surge in gonadal hormones during the first six months of life, known as mini-puberty.^62^ This early hormonal surge coincides exactly with the critical window of gut microbiota assembly identified in our cohort, potentially initiating these dimorphic immunological and metabolic trajectories early in life.^63^ Conversely, *E. lenta*, which is enriched in metabolic disorders, can promote lipopolysaccharide-mediated TLR4 activation and Th17 responses. Beyond these inflammatory pathways, *E. lenta* is uniquely implicated in bile acid metabolism, which directly alters host fat absorption and metabolic homoeostasis.^64^ Crucially, its recognised capacity to metabolise and produce steroid hormones within the gut lumen^65^ provides a direct physiological mechanism for the “microgenderome”. This endocrine-modulating capability may explain the pronounced sex-dependency of the COOC phenotype observed in our cohort, underscoring the need for future strain-level metagenomic investigations to fully elucidate these pathways.

The interpretation of our findings is strengthened by several features of the study design and analytical framework. First, the prospective, longitudinal design of the multi-centre VDAART birth cohort enabled us to examine BMI development dynamically across multiple developmental stages from ages 2–8 years, providing a more comprehensive view of childhood adiposity than a single cross-sectional anthropometric assessment. Second, the availability of detailed maternal, perinatal, and child-level metadata enabled us to evaluate iCD specifically while adjusting for a broad set of potential confounders. Third, by integrating infant gut microbiome profiling with longitudinal BMI trajectories, we examined early-life microbial signatures in relation to subsequent metabolic development within a temporally informative framework. Finally, our use of machine-learning prediction, together with GMPT, provided complementary analytical perspectives, enabled us to move beyond single-association testing and to identify microbiome features that may help contextualise the relationship among delivery mode, gut microbiota, and childhood BMI development.

While our findings suggest a link between the gut microbiota of female children and an increased risk of childhood overweight/obesity in those born via iCD, there are several limitations in our study. A larger sample size or an independent cohort is still warranted to validate our conclusions. Specifically, because the VDAART study is an intervention cohort enriched for maternal asthma risk, validation in broader, observational populations is ideal. However, our comprehensive covariate analyses confirmed that neither maternal Vitamin D supplementation nor maternal asthma status independently confounded the primary association between iCD and long-term BMI development. Another notable limitation is the lack of detailed data on intrapartum antibiotic exposure, which is known to greatly influence the neonatal microbiome. While we excluded stool samples if postnatal antibiotics were used within the 7 days before collection, we could not specifically differentiate vaginal deliveries based on intrapartum antibiotic use. However, since prophylactic antibiotics are standard practice for nearly all CDs, the finding of a significantly higher BMI trajectory exclusively in the iCD group indicates that intrapartum antibiotic exposure alone is not enough to fully explain the increased risk of childhood overweight/obesity. This emphasises the unique and possibly independent effect of the physiological stress associated with iCD. Furthermore, our study is limited by unmeasured nutritional confounding. Future studies with highly granular dietary logs are needed to fully untangle these intersecting pathways. Additionally, our microbiota profiling relies on the 16S rRNA V4 hypervariable region. While we utilised exact ASVs to maximise resolution, the V4 region has inherent limitations in distinguishing certain closely related bacteria at the species level. Therefore, we intend to incorporate multi-omics approaches to gain a more comprehensive understanding of the underlying biological processes in our future work. Nevertheless, our analysis not only establishes an association but also proposes a potential causal relationship between the gut microbiome and childhood overweight/obesity in children born via iCD. Moreover, by identifying inhibitors of COOC, we provide a theoretical basis for the development of future diagnostic and therapeutic strategies in clinical practice.

## Contributors

ZS, JEC, and YYL designed the project. AAL and STW conceptualised and designed the overarching VDAART clinical trial and oversaw the acquisition of longitudinal clinical data, biospecimen collection, and participant follow-up. ZS and TW analysed all the data. ZS and YYL accessed and verified the underlying data. ZS, JAL, JEC, STW, and YYL interpreted the results. ZS wrote the manuscript. JEC and YYL edited the manuscript. JAL, AAL, and STW critically reviewed and revised the manuscript for important intellectual content. All authors approved the manuscript. YYL and JEC supervised the study. All authors read and approved the final version of the manuscript.

## Data sharing statement

Microbiota sequencing data from VDAART are part of the ECHO consortium, and ECHO consortium members can obtain the data directly from the ECHO DCC. For researchers not part of ECHO, de-identified data and data dictionaries are available upon reasonable request to the corresponding author (yyl@channing.harvard.edu), subject to the approval of a brief research proposal and a signed Data Use Agreement to ensure participant privacy. All custom code used for analysis and figure generation in this study has been made publicly available on GitHub (https://github.com/sunzhengCDNM/VDAART_BMI) and archived on Zenodo (https://doi.org/10.5281/zenodo.20417464). Please note that due to participant privacy protections, these public repositories contain only the analytical scripts; the underlying raw datasets must be requested via the formal channel outlined above. Additionally, the GMPT computational tool utilised in this study is publicly available on Zenodo (https://doi.org/10.5281/zenodo.8193075).

## Declaration of interests

JAL reports NIH grants R01HL123915, R01HL15574, and R01HL141826; sponsored research agreements with TruDiagnostic and TOTO; and consulting fees from TruDiagnostic. AAL reports NIH funding for the present manuscript, royalties from UpToDate, and honoraria for service as a DSMB member for the NIH PreCise Network and as DSMB Chair for NIH MICA. STW reports payments from UpToDate. JEC reports NIH support for the present manuscript; grants from the NIH and the Food and Drug Administration paid to his institution; royalties from Harvard Health Publications; consulting fees from the University of Arizona and the American Association for the Advancement of Science; honoraria from Johns Hopkins University School of Medicine, the Academy of Nutrition and Dietetics, the University of Nebraska–Lincoln, Maine Medical Centre, Tufts University, the NIH, the American Society for Reproductive Medicine, the British Dietetic Association, the Pacific Coast Reproductive Society, the University of California San Francisco, George Mason University, and the University of Michigan Ann Arbor; travel support from Johns Hopkins University School of Medicine, National University of Singapore, University of Nebraska–Lincoln, Pacific Coast Reproductive Society, the European Society for Human Reproduction and Embryology, the Foundation for Reproductive Medicine, George Mason University, the British Fertility Society/Society for Reproduction and Fertility/Association of Reproductive and Clinical Scientists, the National Institute of Environmental Health Sciences, the Environmental Mutagenesis and Genomics Society, the Brazilian Urology Society, and Gunma University; participation on Scientific Advisory Boards for Doveras Inc., EPIC-Oxford, and the SPARK Autism Research Study; and stock options in Doveras Inc. All other authors declare no competing interests.
